# Large-scale genome-wide analyses with proteomics integration reveal novel loci and biological insights into frailty

**DOI:** 10.1038/s43587-025-00925-y

**Published:** 2025-08-05

**Authors:** Jonathan K. L. Mak, Chenxi Qin, Moritz Krüger, Anna Kuukka, FinnGen, Aarno Palotie, Mark Daly, Bridget Riley-Gillis, Howard Jacob, Dirk Paul, Slavé Petrovski, Heiko Runz, Sally John, George Okafo, Robert Plenge, Joseph Maranville, Mark McCarthy, Margaret G. Ehm, Kirsi Auro, Simonne Longerich, Anders Mälarstig, Katherine Klinger, Clément Chatelain, Matthias Gossel, Karol Estrada, Robert Graham, Dawn Waterworth, Chris O´Donnell, Nicole Renaud, Tomi P. Mäkelä, Jaakko Kaprio, Petri Virolainen, Antti Hakanen, Terhi Kilpi, Markus Perola, Jukka Partanen, Anne Pitkäranta, Taneli Raivio, Jani Tikkanen, Raisa Serpi, Tarja Laitinen, Veli-Matti Kosma, Jari Laukkanen, Marco Hautalahti, Outi Tuovila, Raimo Pakkanen, Jeffrey Waring, Fedik Rahimov, Ioanna Tachmazidou, Chia-Yen Chen, Zhihao Ding, Marc Jung, Hanati Tuoken, Shameek Biswas, Rion Pendergrass, David Pulford, Neha Raghavan, Adriana Huertas-Vazquez, Jae-Hoon Sul, Xinli Hu, Åsa Hedman, Manuel Rivas, Ma´en Obeidat, Jonathan Chung, Jonas Zierer, Mari Niemi, Samuli Ripatti, Johanna Schleutker, Mikko Arvas, Olli Carpén, Reetta Hinttala, Johannes Kettunen, Arto Mannermaa, Katriina Aalto-Setälä, Mika Kähönen, Johanna Mäkelä, Reetta Kälviäinen, Valtteri Julkunen, Hilkka Soininen, Anne Remes, Mikko Hiltunen, Jukka Peltola, Minna Raivio, Pentti Tienari, Juha Rinne, Roosa Kallionpää, Juulia Partanen, Adam Ziemann, Nizar Smaoui, Anne Lehtonen, Susan Eaton, Sanni Lahdenperä, Natalie Bowers, Edmond Teng, Fanli Xu, Laura Addis, John Eicher, Qingqin S. Li, Karen He, Ekaterina Khramtsova, Martti Färkkilä, Jukka Koskela, Sampsa Pikkarainen, Airi Jussila, Katri Kaukinen, Timo Blomster, Mikko Kiviniemi, Markku Voutilainen, Tim Lu, Linda McCarthy, Amy Hart, Meijian Guan, Jason Miller, Kirsi Kalpala, Melissa Miller, Kari Eklund, Antti Palomäki, Pia Isomäki, Laura Pirilä, Oili Kaipiainen-Seppänen, Johanna Huhtakangas, Nina Mars, Apinya Lertratanakul, Coralie Viollet, Marla Hochfeld, Jorge Esparza Gordillo, Fabiana Farias, Nan Bing, Margit Pelkonen, Paula Kauppi, Hannu Kankaanranta, Terttu Harju, Riitta Lahesmaa, Hubert Chen, Joanna Betts, Rajashree Mishra, Majd Mouded, Debby Ngo, Teemu Niiranen, Felix Vaura, Veikko Salomaa, Kaj Metsärinne, Jenni Aittokallio, Jussi Hernesniemi, Daniel Gordin, Juha Sinisalo, Marja-Riitta Taskinen, Tiinamaija Tuomi, Timo Hiltunen, Amanda Elliott, Mary Pat Reeve, Sanni Ruotsalainen, Audrey Chu, Dermot Reilly, Mike Mendelson, Jaakko Parkkinen, Tuomo Meretoja, Heikki Joensuu, Johanna Mattson, Eveliina Salminen, Annika Auranen, Peeter Karihtala, Päivi Auvinen, Klaus Elenius, Esa Pitkänen, Relja Popovic, Margarete Fabre, Jennifer Schutzman, Diptee Kulkarni, Alessandro Porello, Andrey Loboda, Heli Lehtonen, Stefan McDonough, Sauli Vuoti, Kai Kaarniranta, Joni A. Turunen, Terhi Ollila, Hannu Uusitalo, Juha Karjalainen, Mengzhen Liu, Stephanie Loomis, Erich Strauss, Hao Chen, Kaisa Tasanen, Laura Huilaja, Katariina Hannula-Jouppi, Teea Salmi, Sirkku Peltonen, Leena Koulu, David Choy, Ying Wu, Pirkko Pussinen, Aino Salminen, Tuula Salo, David Rice, Pekka Nieminen, Ulla Palotie, Maria Siponen, Liisa Suominen, Päivi Mäntylä, Ulvi Gursoy, Vuokko Anttonen, Kirsi Sipilä, Hannele Laivuori, Venla Kurra, Laura Kotaniemi-Talonen, Oskari Heikinheimo, Ilkka Kalliala, Lauri Aaltonen, Varpu Jokimaa, Marja Vääräsmäki, Outi Uimari, Laure Morin-Papunen, Maarit Niinimäki, Terhi Piltonen, Katja Kivinen, Elisabeth Widen, Taru Tukiainen, Niko Välimäki, Eija Laakkonen, Jaakko Tyrmi, Heidi Silven, Eeva Sliz, Riikka Arffman, Susanna Savukoski, Triin Laisk, Natalia Pujol, Janet Kumar, Iiris Hovatta, Erkki Isometsä, Hanna Ollila, Jaana Suvisaari, Antti Mäkitie, Argyro Bizaki-Vallaskangas, Sanna Toppila-Salmi, Tytti Willberg, Elmo Saarentaus, Antti Aarnisalo, Elisa Rahikkala, Kristiina Aittomäki, Fredrik Åberg, Mitja Kurki, Aki Havulinna, Juha Mehtonen, Priit Palta, Shabbeer Hassan, Pietro Della Briotta Parolo, Wei Zhou, Mutaamba Maasha, Susanna Lemmelä, Aoxing Liu, Arto Lehisto, Andrea Ganna, Vincent Llorens, Henrike Heyne, Joel Rämö, Rodos Rodosthenous, Satu Strausz, Tuula Palotie, Kimmo Palin, Javier Garcia-Tabuenca, Harri Siirtola, Tuomo Kiiskinen, Jiwoo Lee, Kristin Tsuo, Kati Kristiansson, Kati Hyvärinen, Jarmo Ritari, Katri Pylkäs, Minna Karjalainen, Tuomo Mantere, Eeva Kangasniemi, Sami Heikkinen, Nina Pitkänen, Samuel Lessard, Lila Kallio, Tiina Wahlfors, Eero Punkka, Sanna Siltanen, Teijo Kuopio, Anu Jalanko, Huei-Yi Shen, Risto Kajanne, Mervi Aavikko, Helen Cooper, Denise Öller, Rasko Leinonen, Henna Palin, Malla-Maria Linna, Masahiro Kanai, Zhili Zheng, L. Elisa Lahtela, Mari Kaunisto, Elina Kilpeläinen, Timo P. Sipilä, Oluwaseun Alexander Dada, Awaisa Ghazal, Anastasia Kytölä, Rigbe Weldatsadik, Kati Donner, Anu Loukola, Päivi Laiho, Tuuli Sistonen, Essi Kaiharju, Markku Laukkanen, Elina Järvensivu, Sini Lähteenmäki, Lotta Männikkö, Regis Wong, Auli Toivola, Minna Brunfeldt, Hannele Mattsson, Sami Koskelainen, Tero Hiekkalinna, Teemu Paajanen, Shuang Luo, Shanmukha Sampath Padmanabhuni, Marianna Niemi, Mika Helminen, Tiina Luukkaala, Iida Vähätalo, Jyrki Tammerluoto, Sarah Smith, Tom Southerington, Petri Lehto, Sara Hägg, Jake Lin, Juulia Jylhävä

**Affiliations:** 1 Department of Medical Epidemiology and Biostatistics, Karolinska Institutet, Stockholm, Sweden; 2 Department of Pharmacology and Pharmacy, Li Ka Shing Faculty of Medicine, The University of Hong Kong, Hong Kong, China; 3 Faculty of Medicine and Health Technology and Gerontology Research Center (GEREC), Tampere University, Tampere, Finland; 4 Tampere Institute for Advanced Study, Tampere, Finland; 5 Institute for Molecular Medicine Finland (FIMM), HiLIFE, University of Helsinki, Helsinki, Finland; 6 Broad Institute of MIT and Harvard; Massachusetts General Hospital, Cambridge, MA USA; 7 Abbvie, Chicago, IL USA; 8 AstraZeneca, Cambridge, UK; 9 Biogen, Cambridge, MA USA; 10 Boehringer Ingelheim, Ingelheim am Rhein, Germany; 11 Bristol Myers Squibb, New York, NY USA; 12 Genentech, San Francisco, CA USA; 13 GlaxoSmithKline, Collegeville, PA USA; 14 GlaxoSmithKline, Espoo, Finland; 15 Merck, Kenilworth, NJ USA; 16 Pfizer, New York, NY USA; 17 Translational Sciences, Sanofi R&D, Framingham, MA USA; 18 Maze Therapeutics, San Francisco, CA USA; 19 Janssen Research & Development, LLC, Spring House, <City>, PA USA; 20 Novartis Institutes for BioMedical Research, Cambridge, MA USA; 21 HiLIFE, University of Helsinki, Finland, Finland; 22 Auria Biobank/University of Turku/Hospital District of Southwest Finland, Turku, Finland; 23 THL Biobank/Finnish Institute for Health and Welfare (THL), Helsinki, Finland; 24 Finnish Red Cross Blood Service/Finnish Hematology Registry and Clinical Biobank, Helsinki, Finland; 25 Helsinki Biobank/Helsinki University and Hospital District of Helsinki and Uusimaa, Helsinki, Finland; 26 Northern Finland Biobank Borealis/University of Oulu/Northern Ostrobothnia Hospital District, Oulu, Finland; 27 Finnish Clinical Biobank Tampere/University of Tampere/Pirkanmaa Hospital District, Tampere, Finland; 28 Biobank of Eastern Finland/University of Eastern Finland/Northern Savo Hospital District, Kuopio, Finland; 29 Central Finland Biobank/University of Jyväskylä/Central Finland Health Care District, Jyväskylä, Finland; 30 FINBB - Finnish Biobank Cooperative, Turku, Finland; 31 Business Finland, Helsinki, Finland; 32 GlaxoSmithKline, Stevenage, UK; 33 University of Stanford, Stanford, CA USA; 34 Faculty of Medicine and Health Technology, Tampere University, Tampere, Finland; 35 Northern Savo Hospital District, Kuopio, Finland; 36 Northern Ostrobothnia Hospital District, Oulu, Finland; 37 University of Eastern Finland, Kuopio, Finland; 38 Pirkanmaa Hospital District, Tampere, Finland; 39 Hospital District of Helsinki and Uusimaa, Helsinki, Finland; 40 Hospital District of Southwest Finland, Turku, Finland; 41 Institute for Molecular Medicine Finland, HiLIFE, University of Helsinki, Helsink, Finland; 42 GlaxoSmithKline, Brentford, UK; 43 Janssen Research & Development, LLC, Titusville, NJ USA; 44 University of Gothenburg, Gothenburg, Sweden; 45 Seinäjoki Central Hospital, Seinäjoki, Finland; 46 Tampere University, Tampere, Finland; 47 Novartis, Basel, Switzerland; 48 Finnish Institute for Health and Welfare (THL), Helsinki, Finland; 49 Broad Institute, Cambridge, MA USA; 50 Massachusetts General Hospital, Boston, MA USA; 51 Janssen Research & Development, LLC, Boston, MA USA; 52 Novartis, Boston, MA USA; 53 Janssen-Cilag Oy, Espoo, Finland; 54 Department of Molecular Genetics, University of Lodz, Lodz, Poland; 55 Helsinki University Hospital and University of Helsinki, Helsinki, Finland; 56 Eye Genetics Group, Folkhälsan Research Center, Helsinki, Finland; 57 Research Unit of Oral Health Sciences Faculty of Medicine, University of Oulu, Oulu, Finland; 58 Medical Research Center, Oulu, Oulu University Hospital and University of Oulu, Oulu, Finland; 59 University of Helsinki, Helsinki, Finland; 60 University of Jyväskylä, Jyväskylä, Finland; 61 University of Oulu, Oulu, Finland; 62 Estonian biobank, Tartu, Estonia; 63 Department of Otorhinolaryngology - Head and Neck Surgery, University of Helsinki and Helsinki University Hospital, Helsinki, Finland; 64 University of Eastern Finland and Kuopio University Hospital, Department of Otorhinolaryngology, Kuopio, Finland; 65 Department of Allergy, Helsinki University Hospital and University of Helsinki, Helsinki, Finland; 66 Department of Medical Genetics, Helsinki University Central Hospital, Helsinki, Finland; 67 Transplantation and Liver Surgery Clinic, Helsinki University Hospital, Helsinki University, Helsinki, Finland; 68 University of Helsinki and Hospital District of Helsinki and Uusimaa, Helsinki, Finland; 69 University of Tampere, Tampere, Finland; 70 Finnish Red Cross Blood Service, Helsinki, Finland; 71 European Molecular Biology Laboratory, European Bioinformatics Institute, Cambridge, UK; 72 Finnish Biobank Cooperative - FINBB, Turku, Finland

**Keywords:** Genome-wide association studies, Systems biology, Diseases, Ageing

## Abstract

Frailty is a clinically relevant phenotype with notable gaps in our understanding of its etiology. Using the Hospital Frailty Risk Score (HFRS) to define frailty, we performed a genome-wide association study in FinnGen (*N* = 500,737), replicated the results in the UK Biobank (*N* = 407,463) and performed a meta-analysis. We prioritized genes through colocalization with expression, splicing and protein quantitative trait loci and proteomics integration. We identified 53 independent lead variants associated with frailty (*P* < 5 × 10^−8^), of which 45 were novel and not previously reported in the GWAS Catalog. Replication at the individual variant and polygenic risk score of the HFRS (*P* = 1.86 × 10^−522^) levels and meta-analysis largely confirmed the findings. Colocalization analysis supported a causal role for several genes, including *CHST9*, *C6orf106* (*ILRUN*), *KHK*, *MET*, *APOE*, *CGREF1* and *PPP6C*. Additionally, plasma levels of MET, CGREF1 and APOE were associated with HFRS. Our results reveal new genetic contributions to frailty and shed light on its biological basis.

## Abstract (web summary)

To better understand the etiology of frailty, the authors perform a large genetic study. They identified 45 additional variants and implicated *MET*, *CHST9*, *ILRUN*, *APOE*, *CGREF1* and *PPP6C* as potential causal genes, linking frailty to immune regulation, metabolism and cellular signaling.

## Main

Aging is a highly complex process with substantial heterogeneity in health trajectories among individuals. Frailty represents a clinically relevant aging phenotype that gauges health in aging^1^ and predicts various adverse outcomes independent of chronological age^2^. Frailty describes a syndrome of decreased physiological reserves across multiple homeostatic systems^1^. Currently, no gold standard exists to measure frailty; instead, several scales with different properties have been developed, each capturing partially different at-risk populations^3^. Created based on 109 weighted International Classification of Diseases, 10th Revision (ICD-10) codes characterizing older adults with high resource use and diagnoses associated with frailty, the HFRS presents a relatively new scale to measure frailty^4^. It has a fair overlap with existing frailty definitions based on the deficit accumulation (frailty index (FI)) and phenotypic (frailty phenotype (FP)) models of frailty and has a moderate agreement with the FI^4^. While the HFRS uses ICD-10 codes for administrative ease, enabling the measurement of frailty in real-world data, the FI^5^ and FP^6^ are rooted in clinical and functional data and are often assessed in cohort studies. The FI is a multidimensional measure of frailty, offering a comprehensive view of a person’s overall health^5^. In contrast, the FP defines frailty through specific physical characteristics: weakness, slowness, exhaustion, low physical activity and weight loss^6^. While each measure captures distinct aspects of frailty, together they provide a more complete understanding of the condition.

The etiology of frailty remains incompletely understood. Twin studies by us and others suggest that frailty, measured using the FI, is up to 52% heritable^7,8^, with relatively stable genetic influences across age^9^. To date, only two previous large-scale genome-wide association studies (GWASs) of frailty exist. Atkins et al. performed a meta-analysis GWAS of FI that identified 34 loci and estimated the single nucleotide polymorphism (SNP) heritability of the FI at 11%^10^. Ye at al. identified 123 loci for FP and estimated the SNP heritability of the FP at 6%^11^. However, it is likely that additional genetic signals exist and analyses in other large populations can shed further light on the genetic underpinnings of frailty.

To date, no previous studies into the genetics of frailty using the HFRS exist. To this end, we performed a GWAS of the HFRS in FinnGen (*N* = 500,737), with replication of the results in the UK Biobank (*N* = 407,463), both at the individual variant level and through polygenic risk scores (PRSs). We also performed a meta-analysis on the results from both GWASs to capture the totality of the evidence. Given that dementia has the highest weight in the HFRS definition, we performed a sensitivity analysis by excluding dementia from the HFRS definition and similarly replicated the results in the UK Biobank and conducted a meta-analysis on the results. A functional follow-up to identify causal genetic loci was performed through colocalization analysis^12^ with expression, splicing and protein quantitative trait loci (eQTL, sQTL and pQTL, respectively) and associating measured protein levels with the HFRS in the UK Biobank (*N* = up to 42,495).

## Results

### Sample characteristics

**Fig. 1:**
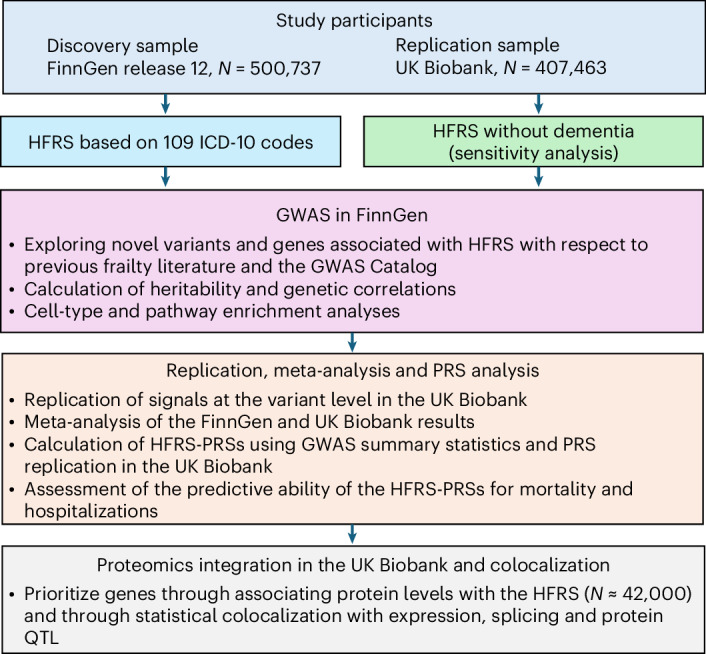
Outline of the study. Discovery GWASs of HFRS and HFRS without dementia were performed in FinnGen to identify genetic variants associated with frailty. The significant variants (*P* ≺ 5 × 10^−8^) were then replicated in the UK Biobank, and a meta-analysis of the FinnGen and UK Biobank results was performed. The GWAS summary statistics of FinnGen were used to calculate HFRS-PRSs, which were then assessed for their association with mortality and hospitalizations in the UK Biobank. Finally, protein association and colocalization analyses were performed to prioritize genes and identify causal variants.

The workflow of the analyses is presented in Fig. 1. In the HFRS GWAS, we included 500,737 (282,202 females, 56.4%) FinnGen and 407,463 UK Biobank participants (220,208 females, 54.1%). Characteristics of the study populations are presented in Table 1.

**Table 1:** Characteristics of the study samples

Characteristic	FinnGen	UK Biobank
No. of individuals	519,200	407,463
Age at baseline assessment, mean (s.d.)	53.1 (17.9)	56.9 (8.0)
Age at end of follow-up/death, mean (s.d.)	60.8 (18.0)	70.9 (7.9)
Sex, *n* (%)		
Women	292,784 (56.4)	220,208 (54.1)
Men	226,416 (43.6)	187,255 (45.9)
BMI (kg/m^2^), mean (s.d.)	27.35 (5.53)	27.41 (4.76)
Missing, *n* (%)	142,454 (27.4)	1273 (0.3)
Smoking, *n* (%)		
Nonsmoker	156,355 (50.9)	221,770 (54.6)
Former smoker	70,317 (22.9)	143,384 (35.3)
Current smoker	80,736 (26.2)	41,109 (10.1)
Missing	211,792	1,380
HFRS, median (IQR)	5.2 (1.6–10.4)	1.5 (0–5)
Women, median (IQR)	5.3 (1.6–10.5)	1.5 (0–4.7)
Men, median (IQR)	5.0 (1.5–10.3)	1.5 (0–5.4)
HFRS categories, *n* (%)		
Low risk (<5)	241,656 (48.4)	304,555 (74.7)
Intermediate risk (5–15)	188,147 (37.8)	74,386 (18.2)
High risk (>15)	65,925 (13.2)	28,702 (7.0)
HFRS > 5, *n* (%)	254,874 (51.0)	101,326 (24.9)
HFRS > 5 before age 65, *n* (%)	95,410 (18.4)	33,485 (8.2)
Died during follow-up, *n* (%)	62,764 (12.1)	36,795 (9.0)
Number of hospitalizations, median (IQR)	8 (4–17)	1 (0–3)

FinnGen participant characteristics are presented for the sample with non-missing data on the HFRS (*N* = 519,200).

IQR, interquartile range.

### Discovery GWAS of HFRS in FinnGen

**Fig. 2:**
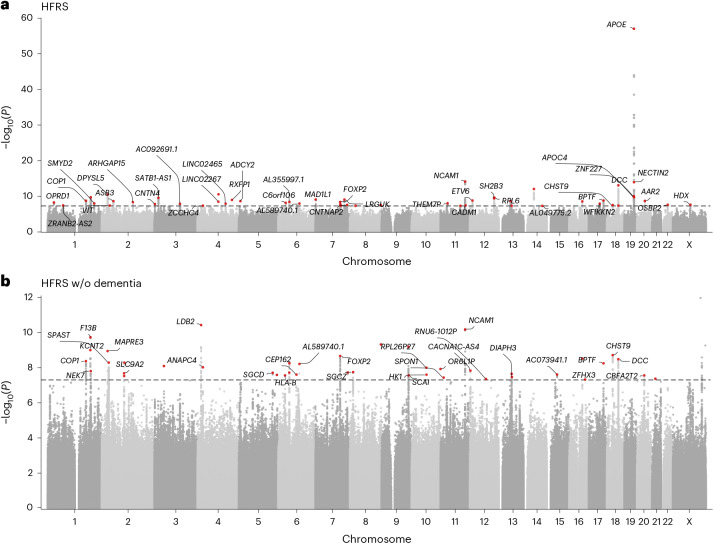
GWAS results in FinnGen. **a**,**b**, Manhattan plots for the associations with HFRS (**a**) and HFRS excluding dementia (**b**) in FinnGen using linear mixed-effects modeling adjusted for birth year, sex and the first ten PCs. The dashed lines indicate the genome-wide significance threshold (*P* = 5 × 10^−8^). The annotations represent the independent lead variants associated with frailty.

**Fig. 3:**
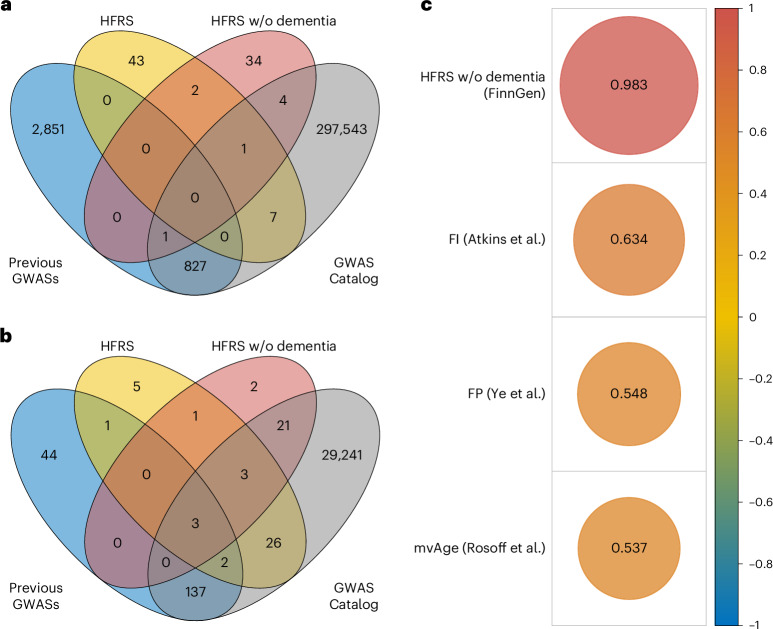
Lead variants and genes and genetic correlations of the HFRS. **a**, Venn diagram showing the overlap of the lead variants associated with the full HFRS and the HFRS without dementia in FinnGen and those reported in the literature. Previous GWASs refers to genes identified in for the FI^10^, FP^11^ and mvAge^13^. **b**, Venn diagram showing the overlap of the lead variant genes associated with the full HFRS and the HFRS without dementia in FinnGen and those reported in the literature. **c**, Genetic correlations between HFRS in FinnGen and other frailty-related traits estimated using linkage disequilibrium score regression. All the correlations were statistically significant at *P* < 2.2 × 10^−16^.

We identified 1,588 variants associated (*P* < 5 × 10^−8^) with the HFRS in the main analysis and 492 variants in the sensitivity analysis, which removed the dementia weights from the HFRS (Fig. 2a,b and Supplementary Tables 1 and 2). Of these, 53 variants (at 50 loci) and 42 variants (at 42 loci) were identified as independent lead variants (*r*^2^ < 0.01) for the HFRS and HFRS without dementia, respectively. As dementia diagnosis has the highest weight in the HFRS formula, the most influential peak expectedly resided in the *APOE* (rs7412) region on chromosome 19 (Fig. 2a). Sensitivity analysis confirmed the expected loss of the *APOE* peak (Fig. 2b). Of the independent lead variants associated with HFRS and HFRS without dementia, 45/53 and 36/42, respectively, were novel with respect to the GWAS Catalog and previously reported GWAS results of the FI^10^, FP^11^ and mvAge^13^ (Fig. 3a and Supplementary Tables 1 and 2). The variants mapped to 41 (HFRS) and 30 (HFRS without dementia) genes of which 6 and 3, respectively, were novel, that is, previously unreported for any trait at *P* < 5 × 10^−8^. The results also demonstrated unique, non-shared associations in both analyses (Fig. 3b and Supplementary Tables 1 and 2). Supplementary Table 3 presents the shared and unique genes between the HFRS, FI and FP GWASs.

### Replication in the UK Biobank and meta-analysis

For HFRS, 1,262/1,588 variants were available for replication and meta-analysis. In the UK Biobank, 73 variants (6%) replicated at *P* < 5 × 10^−8^ and 688 (55%) at *P* < 0.05, while in the meta-analysis, 357 variants (28%) replicated at *P* < 5 × 10^−8^ and 1,260 (100%) at *P* < 0.05 (Supplementary Table 1). Of the 53 lead variants, 36 were available; 2 lead variants (6%) replicated at *P* < 5 × 10^−8^ and 14 (39%) at *P* < 0.05 in the UK Biobank, while 6 (17%) replicated at *P* < 5 × 10^−8^ and 35 (97%) at *P* < 0.05 in the meta-analysis (Supplementary Table 1). For HFRS without dementia, 435/492 variants were available for replication and meta-analysis. In the UK Biobank, 21 variants (5%) replicated at *P* < 5 × 10^−8^ and 118 (27%) at *P* < 0.05, while in the meta-analysis, 50 variants (11%) replicated at *P* < 5 × 10^−8^ and 435 (100%) at *P* < 0.05 (Supplementary Table 1). Of the 42 lead variants, 26 were available; 1 lead variant (4%) replicated at *P* < 5 × 10^−8^ and 10 (38%) at *P* < 0.05 in the UK Biobank, while 4 (17%) replicated at *P* < 5 × 10^−8^ and 26 (100%) at *P* < 0.05 in the meta-analysis (Supplementary Table 2). The effect direction was consistent for all variants that replicated at *P* < 5 × 10^−8^ in the meta-analysis (Supplementary Tables 1 and 2).

### Genetic correlation and heritability

**Extended Data Fig. 1:**
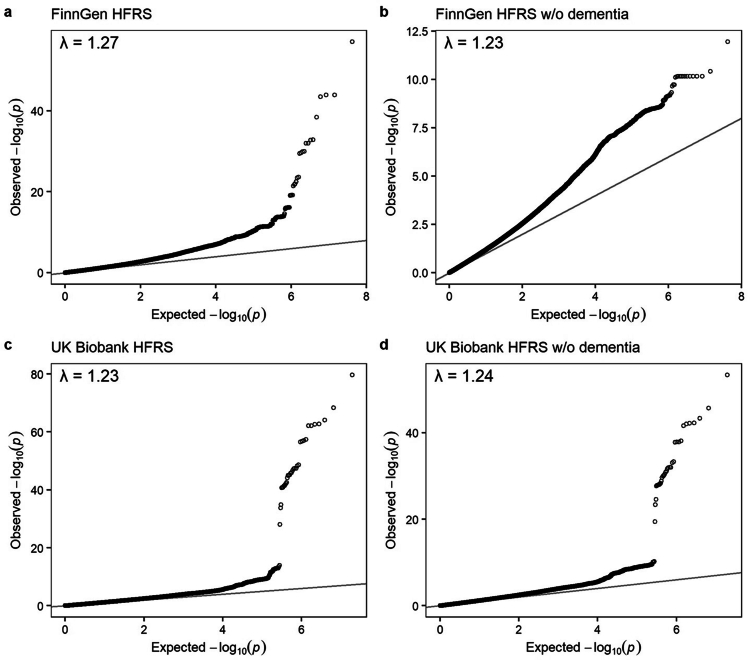
QQ-plots of association summary statistics for the HFRS and HFRS without dementia. Panels **a** and **b** show results for FinnGen, and panels **c** and **d** for the UK Biobank, respectively.

We observed a lambda genomic control value of 1.27 with an intercept of 1.19 (s.e. = 0.011) for HFRS and 1.11 with an intercept of 1.23 (s.e. = 0.010) for HFRS without dementia (QQ plots provided in Extended Data Fig. 1). Despite the relatively high lambda values, the intercepts suggest that the inflation in test statistics was mainly due to polygenicity, rather than bias due to population stratification. The SNP heritability was 0.06 (s.e. = 0.002) for HFRS and 0.04 (s.e. = 0.002) for HFRS without dementia. Statistically significant and positive genetic correlations (*P* < 2.2 × 10^−16^) were observed between HFRS and previous GWASs on frailty and mvAge (Fig. 3c).

### Cell-type and pathway enrichment

**Extended Data Fig. 2:**
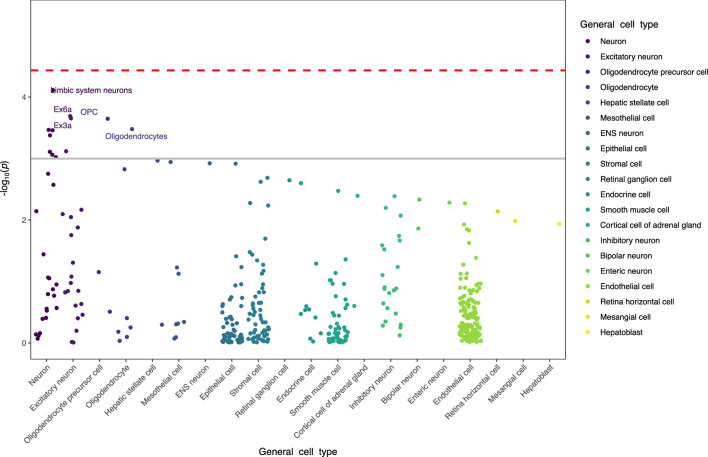
Cell-type enrichment analysis of HFRS. Top 20 enriched cell types for the variants associated with the HFRS in FinnGen are shown.

**Extended Data Fig. 3:**
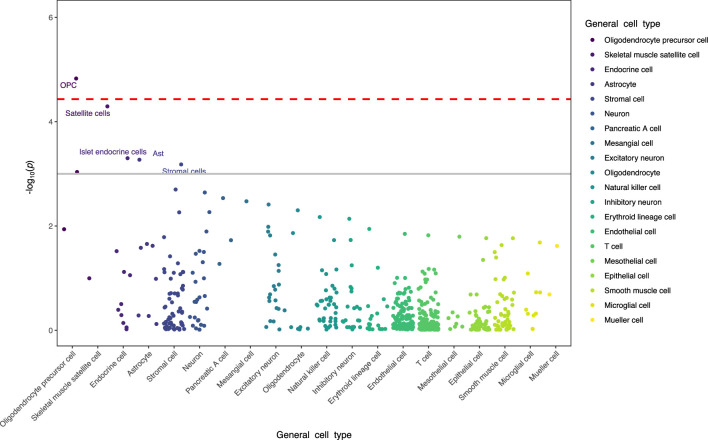
Cell-type enrichment analysis of HFRS without dementia. Top 20 enriched cell types for the variants associated with the HFRS without dementia in FinnGen are shown.

For HFRS, the top (*P* < 3.7 × 10^−5^, corrected for multiple testing) cell types enriched for expression were limbic system neurons in cerebrum, excitatory neurons (Ex6) in visual cortex, oligodendrocyte precursor cells (OPCs) in cerebellar hemisphere and oligodendrocytes in cerebellum (Extended Data Fig. 2 and Supplementary Table 4). For HFRS without dementia, the top cell types were OPCs and astrocytes in cerebellar hemisphere, skeletal muscle satellite cells in muscle and endocrine cells in stromal cells in stomach (Extended Data Fig. 3 and Supplementary Table 5). Enrichr^14^ pathway analysis (adjusted *P* < 0.05) showed that the top pathways for the HFRS signals were relevant to the nervous system functions (herpes simplex virus 1 infection, netrin-mediated repulsion signals), cell adhesion and lipid metabolism (Supplementary Table 6). Comparison of the pathways from the HFRS, FI and FP GWASs revealed overlap in herpes simplex virus 1 infection and cell adhesion molecules between HFRS and FI, and in multiple pathways related to lipid and lipoprotein metabolism, cellular interactions and adhesion between HFRS and FP (Supplementary Table 6). Each GWAS also had distinct pathways not shared with the others (Supplementary Table 6). For HFRS without dementia, several functions related to cell cycle were enriched at *P* < 0.05, although none of the pathways were statistically significant after correction for multiple testing (Supplementary Table 7).

### Exploring causal variants through proteomics integration

**Fig. 4:**
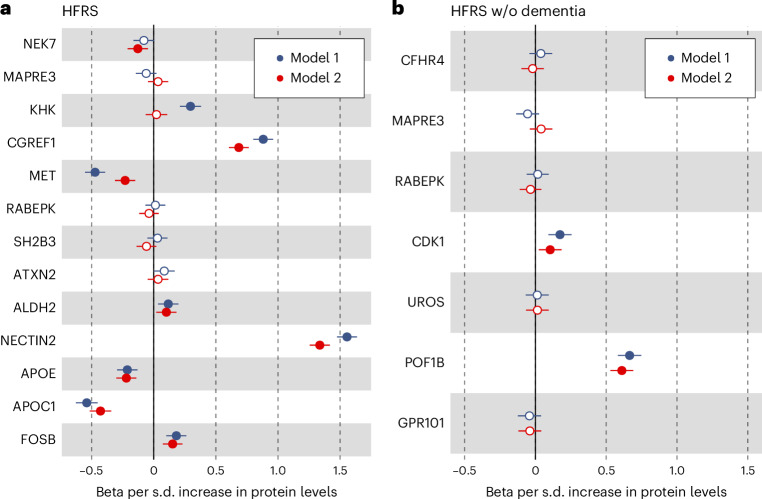
Proteomics integration in the UK Biobank. **a**,**b**, Protein associations (beta coefficients) with the full HFRS (**a**) and HFRS without dementia (**b**) the in the UK Biobank using linear regression models (*N* = 34,879–42,495; exact *N* for each model is given in Supplementary Table 10). All models were adjusted for birth year, sex and the first ten PCs (model 1), and additionally adjusted for batch, baseline assessment center, BMI and smoking (model 2). Solid dots indicate statistically significant associations at an FDR < 0.05. The bars indicate 95% confidence intervals.

To identify potentially causal and functional variants (that is, missense, splice region, loss of function and 5′ and 3′ untranslated region variants associated with the HFRS and HFRS without dementia at *P* < 5 × 10^−7^; Supplementary Tables 8 and 9), we associated the protein levels of the corresponding genes to HFRS (13 proteins available in UK Biobank Olink platform) and HFRS without dementia (8 proteins available in UK Biobank Olink platform). We adjusted the models for birth year, sex and the first ten principal components (PCs; model 1), as well as batch, baseline assessment center, body mass index (BMI) and smoking (model 2). Significantly associated proteins at a false discovery rate (FDR) < 0.05 in both models 1 and 2 were CGREF1, MET, ALDH2, NECTIN2, APOC1, APOE and FOSB for HFRS, and CDK and POF1B for HFRS without dementia (Fig. 4 and Supplementary Table 10).

### Colocalization analysis

**Extended Data Fig. 4:**
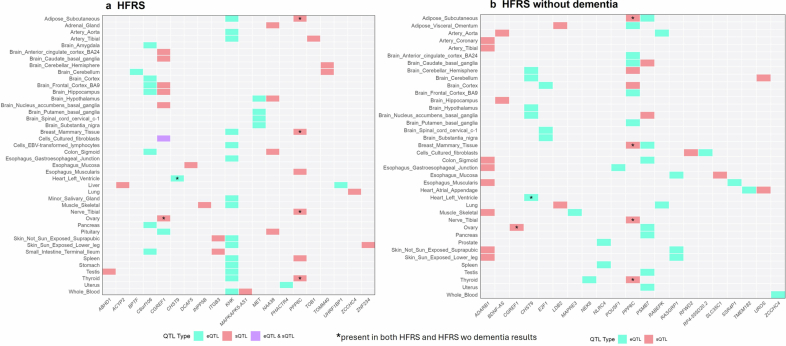
Colocalized expression and splicing quantitative trait loci. Colocalized eQTL and sQTL by tissue with the genes associated with (**a**) HFRS and (**b**) HFRS without dementia. For each gene, the posterior probability for a shared causal variant was >80%.

**Extended Data Fig. 5:**
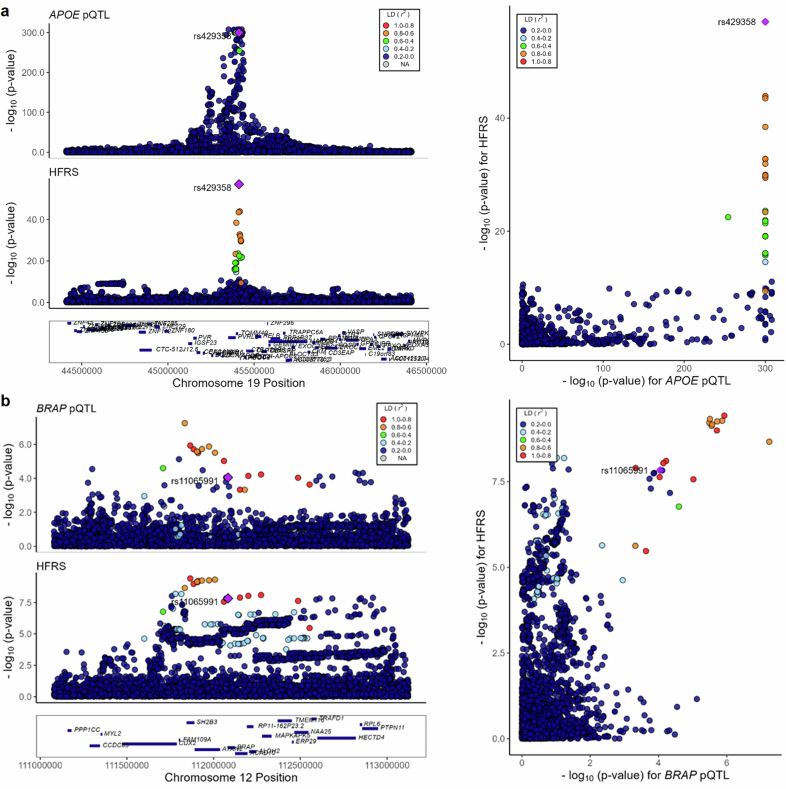
Regional association plots for gene loci. Regional association plots for gene loci – panel **a** for *APOE* and panel **b** for *BRAP* – identified in the colocalization analysis of protein quantitative trait loci (pQTL) and the variants associated with the Hospital Frailty Risk Score (HFRS).

Several gene loci, such as *CHST9*, *C6orf106* (*ILRUN*), *KHK*, *MET*, *CGREF1* and *PPP6C* had shared causal variants (posterior probability for H4 (PP.H4) > 80%) in eQTL and/or sQTL for HFRS. Several colocalized (PP.H4 > 80%) eQTL and/or sQTL loci were also identified for HFRS without dementia, including *CHST9*, *CGREF1*, *PPP6C*, *ADARB1* and *PSMB7*. The full eQTL and sQTL colocalization results for the HFRS and HFRS without dementia are presented in Supplementary Tables 11 and 12, and the colocalization results with a PP.H4 > 80% are summarized by tissue for each gene in Extended Data Fig. 4. In the pQTL analysis, of those genes that had a protein measurement available (that is, the protein was detectable in plasma), a total of 20 loci for HFRS and 9 loci for HFRS without dementia had enough power for the analyses (PP > 88%; Methods). Of them, a colocalized signal (that is, shared single causal variant, PP.H4 > 80%; Methods) was detected within *APOE* and *BRAP* genes for HFRS (Supplementary Table 13), whereas no colocalized signal with pQTL was detected within genes for HFRS without dementia. For most of the tested genes, the PP.H3 values were greater than or close to 90%, indicative of distinct causal variants for protein levels and HFRS (Supplementary Tables 13 and 14). Regional association plots of the *APOE* gene demonstrated that the strongest signal peak rs429358 and variants in high linkage disequilibrium with it fall in the vicinity (Extended Data Fig. 5).

### HFRS-PRS analyses in the UK Biobank

**Fig. 5:**
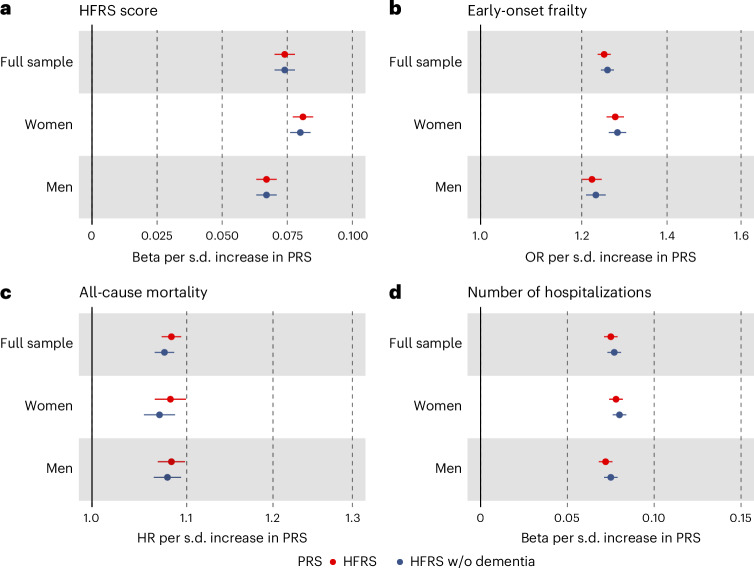
HFRS-PRSs, frailty, mortality, and hospitalizations. **a**–**d**, Associations of the HFRS-PRSs with the HFRS (**a**), early-onset frailty (**b**), all-cause mortality (**c**) and number of hospitalizations (**d**) in the UK Biobank (*N* = 407,463). All models included birth year, birth region, sex and the first ten PCs as covariates. The bars indicate 95% confidence intervals of the beta coefficients, odds ratios (ORs) and hazard ratios (HRs).

The PRSs for HFRS (HFRS-PRSs) were statistically significantly associated with the HFRS in the UK Biobank (*β* = 0.074 per s.d. increase; *P* = 1.86 × 10^−522^) after adjusting for birth year, sex and the first ten PCs (Fig. 5a). Next, using similar adjustments, we analyzed whether the HFRS-PRSs could predict early-onset frailty in the UK Biobank (that is, HFRS > 5 before age 65) and observed an odds ratio of 1.25 (*P* = 2.0 × 10^−322^; Fig. 5b). We further examined whether the HFRS-PRSs could predict all-cause mortality and number of hospitalizations and found statistically significant associations with both outcomes (Fig. 5c,d). The estimates of the HFRS-PRSs were similar in men and women compared to the full sample, and also consistent for the HFRS-PRsS excluding dementia (Fig. 5a–d). Numeric estimates for all the HFRS-PRS analyses are presented in Supplementary Table 15. Lastly, we found that adding the HFRS-PRSs to a model with age, sex and the first ten PCs significantly improved model performance on mortality and hospitalizations, as assessed by likelihood-ratio and *F*-test statistics (Supplementary Table 16).

### Prediction of mortality using HFRS

To assess the validity of HFRS in predicting mortality, we examined its association with all-cause mortality and found that higher HFRSs, both with and without dementia, were associated with mortality in FinnGen (hazard ratio 1.29 for both HFRS and without dementia) and UK Biobank (hazard ratio 1.48 for both HFRS without dementia), independent of age, birth year and sex (Supplementary Table 17).

## Discussion

Our study represents a large GWAS of frailty using the HFRS. We identified 1,588 associated variants and 53 lead variants, of which 45 were novel, and not previously reported for any trait. The lead variants mapped to 41 genes, of which 6 were novel. Replication in the UK Biobank and subsequent meta-analysis showed that 28% of all variants and 17% of lead variants replicated at *P* < 5 × 10^−8^, while 100% of all variants and 97% of lead variants replicated at *P* < 0.05 in the meta-analysis. Colocalization analysis identified several causal candidate genes, including *CHST9*, *C6orf106* (*ILRUN*), *KHK*, *MET*, *APOE*, *CGREF1* and *PPP6C*. Additionally, plasma levels of MET, CGREF1 and APOE were associated with HFRS, further supporting their roles in frailty. We also derived PRSs for HFRS and showed that they predict frailty, early-onset frailty, mortality and hospitalizations in the UK Biobank.

The strongest GWAS signals were observed in the *TOMM40*/*APOE*/*APOC1*/*NECTIN2* locus on 19q13.3, a locus in strong linkage disequilibrium and known for its associations with cognitive^15^ and cardiometabolic^16^ traits. We observed the strongest signal for the missense variant rs429358 (388 T > C) that, together with rs7412, defines the *APOE* ε2, ε3 and ε4 haplotypes. The rs7412 was, however, not associated with frailty in our study. A similar finding has been observed for longitudinal weight loss—a feature that also characterizes frailty—where rs429358 increased the risk, while rs7412 did not^17^. Our sensitivity analysis, which removed dementia from the HFRS, truncated the chromosome 19 peak as expected and revealed additional loci. The HFRS lead variants mapped to 42 genes, 7 of which were shared with HFRS without dementia, while 31 genes were uniquely mapped in HFRS without dementia. The unique lead variant associations for both HFRS and HFRS without dementia are plausible, as dementia had the highest individual weight in the HFRS definition, and for highly polygenic traits like frailty, even small differences in phenotype definitions can influence which variants reach genome-wide significance. Genetic correlation between HFRS and HFRS without dementia was nevertheless almost perfect (0.98), indicating the same underlying genetic construct.

The genes to which the 45 novel lead variants for the HFRS mapped include *C6orf106* (*ILRUN*) and *CHST9*, both of which also displayed colocalized signals with eQTL across different tissues, supporting their potential causal roles. *C6orf106* (*ILRUN*) is a regulator of inflammation and lipid metabolism^18^, while *CHST9* encodes an enzyme essential for cell–cell interactions and signal transduction^19^. Notably, several *CHST9* variants were also associated with HFRS without dementia and similarly exhibited colocalization with eQTL. *CGREF1*, a gene linked to cell cycle regulation and adhesion^20^, and *PPP6C*, a gene involved in nuclear factor-κB pathway regulation^21^, showed the same sQTL-colocalized gene–tissue pairs for HFRS and HFRS without dementia, supporting their functional roles in frailty, irrespective of the HFRS definition. While *C6orf106 (ILRUN)*, *CHST9*, *CGREF1* and *PPP6C* are functionally diverse, they collectively link immunoinflammatory modulation, cellular interactions and adhesion to frailty. Specific to HFRS, we additionally identified multiple colocalized signals in *KHK* and *MET*, while for HFRS without dementia, we identified additional colocalized signals in *ADARB1* and *PSMB7*. Aside from a few links to blood pressure, plasma lipids or BMI in the GWAS Catalog, *CHST9*, *CGREF1*, *PPP6C*, *KHK*, *MET*, *ADARB1* and *PSMB7* have no prior GWAS associations with the HFRS conditions, suggesting that HFRS, as a composite measure, can offer insights into frailty beyond its individual components.

Proteomics integration showed that CGREF1, NECTIN2, MET and APOC1 were associated with the HFRS with the largest effect sizes; elevated levels of the first two and lower levels of the latter two were associated with higher HFRS scores. Previous studies have linked elevated circulating NECTIN2 levels to Alzheimer’s disease risk^22^ and low APOC1 levels to cognitive decline and frailty, as defined using the FP^23^, which likely explains their associations with the HFRS. In contrast, no prior studies have linked plasma CGREF1 or MET to frailty or HFRS conditions, highlighting a novel association. Additionally, as *CGREF1* and *MET* exhibited eQTL-colocalized and/or sQTL-colocalized signals across multiple tissues, their protein-level associations further support their biological relevance in frailty.

We estimated the SNP heritability of HFRS at 6%, an estimate in the same range as previously reported for the FI (11%)^10^ and FP (6%)^11^. Genetic correlations between HFRS, FI and FP were moderate, ranging from 0.54 to 0.63, while gene-level overlap was limited: two shared genes between HFRS and FI and eight between HFRS and FP. The limited gene-level overlap is likely a result of frailty being a highly polygenic trait, where genome-wide significant variants represent only a fraction of the total genetic signal. Genetic correlation, in turn, reflects the combined influence of numerous variants, including those that do not reach the genome-wide significance threshold, but still make a substantial overall contribution to the trait. Moreover, it has been shown that different frailty scales identify only partially overlapping groups of individuals as frail^4,24^, suggesting that these scales may capture somewhat distinct constructs. In our previous study^25^, we assessed the phenotypic correlation between HFRS and FI at 0.21 and HFRS and FP at 0.31 in the UK Biobank participants, indicating somewhat lower phenotypic correlations compared to their genotypic counterparts. A possible explanation is that, because most UK Biobank participants are still relatively young, frailty may not yet be fully expressed, leading to many values being 0 and thereby diluting the phenotypic correlations. Additionally, environmental factors, such as physical activity, may directly influence phenotypic frailty, but might not affect the multidimensional FI or HFRS to the same extent, leading to reduced phenotypic correlations. The overall low prevalence of frailty in the UK Biobank participants may also have contributed to the low gene-level overlap between FI, FP and HFRS because both the FI^10^ and FP^11^ GWASs included UK Biobank participants. For the same reason, the overall lower HFRS scores in the UK Biobank and differences in the proportions of individuals with certain HFRS conditions between FinnGen and the UK Biobank may have also affected the replication results, potentially leading to underestimated effect sizes in the UK Biobank and the overlooking of some true associations.

Cell-type enrichment indicated enriched expression of the genes associated with the signals in various neuronal cells, such as limbic system neurons, excitatory neurons, OPCs and oligodendrocytes located in the cerebrum, visual cortex, cerebellar hemisphere and cerebellum, respectively. Enrichment of OPCs (cerebellar hemisphere) persisted even after removing the contribution of dementia diagnoses from the HFRS. Expression enrichment in brain tissues was likewise observed the GWAS of FI^10^, which identified frontal cortex BA9, cerebellar hemisphere, spinal cord cervical C1 and hippocampus as statistically significant. The GWAS on FP^11^ also identified the genetic signals enriched in brain tissues, such as cerebellar hemisphere, frontal cortex BA9 and cerebellum. It is noteworthy that neither FI nor FP in these GWASs included any items of cognition or dementia diagnosis in the frailty definition. Our findings thus reinforce the role of central nervous system functions in frailty, regardless of the frailty definition.

Our pathway analyses highlighted Herpes simplex virus 1 infection and various cell adhesion and lipid/lipoprotein metabolism pathways relevant to the signals. The first two pathways overlapped with the FI pathways, while lipid metabolism processes were shared with the FP pathways. However, several pathways were unique to each frailty measure: FI was enriched for immunoinflammatory functions, while FP included cardiac and membrane transport processes. These differences likely stem from the varying components of each frailty measure. The HFRS, which includes 109 conditions capturing both multisystem decline and core physiological senescence, showed enrichment in fundamental processes like cell adhesion and lipoprotein metabolism. The FI, also reflecting multisystem decline, appears particularly influenced by immunoinflammatory factors, as seen also in previous associations with GlycA^26^, a marker of systemic inflammation, including studies supporting a causal link^27^. Many FI-related conditions, such as cardiovascular disease and diabetes, also have inflammatory components, potentially explaining the connection. The FP, which mostly focuses on physical frailty, was enriched for cardiac function and membrane transport pathways, both essential for muscle activity, ion flux regulation and nutrient uptake.

To assess the usefulness of the HFRS in our samples, we showed that it predicts mortality independent of sex and birth year and performs equally well even when dementia is excluded. Similarly, the HFRS-PRSs, also when dementia was removed, associated with the risk of frailty, early-onset frailty, mortality and hospitalizations. As frailty manifests relatively late in life for most individuals, risk assessment through PRSs may offer possibilities for early intervention to mitigate frailty before it escalates. Future studies are needed to ascertain the clinical utility of such approaches.

Our definition of frailty was based on clinical diagnoses in register data; such an approach has both advantages and disadvantages. A notable advantage is that in Finland and the United Kingdom, public healthcare is primarily tax-funded, and each citizen has equal access. Issues pertinent to self-reported data, such as recall bias and missing information were also avoided. On the other hand, some conditions may be underreported in the registers, while others may have a lag from the onset of symptoms to assigning the diagnosis. We also note that the genetic associations were weaker in the UK Biobank compared to FinnGen, a finding likely explained by healthy selection due to volunteer-based participation in the UK Biobank^28^ compared to FinnGen, which consists of national cohorts and biobank samples of hospitalized individuals. Also pertinent to all GWASs, the discovery samples tend to have stronger association statistics compared to replication, a phenomenon known as the winner’s curse.

In conclusion, we provide a large GWAS on HFRS and reveal new genetic contributions and causal candidate genes. Overall, the results reinforce previous findings that immunoinflammatory and nervous system functions are relevant to the etiology of frailty, regardless of how frailty is defined. Future studies should thus explore the role of these functions in the development of frailty, including cognitive frailty, to better understand the etiology of frailty.

## Methods

This work complies with all relevant ethical regulations. A full list of the ethics boards that approved the study protocols is provided at the end of this section.

### Samples

FinnGen is a large national genetic resource (*N* = 520,210; release 12) established in 2017 and consists of Finnish individuals, aged 18 years and older at study baseline^29^. FinnGen includes prospective epidemiological and disease-based cohorts, as well as hospital biobank samples. Information on diagnoses since 1969 was linked by the unique national personal identification number to national healthcare, population and cause of death registries and recorded using the ICD Revisions 8–10. Information on dates and causes of death were obtained via linkages to the population and cause of death registers through 30 September 2023 (R12 v1). After excluding individuals with missing information on baseline age, birth year and sex, and samples not passing genotyping quality control (see below), we included 500,737 FinnGen participants in this study.

The UK Biobank includes 502,642 volunteer participants, aged 37 to 73 years old at baseline, recruited through 22 assessment centers across England, Scotland and Wales between 2006 and 2010 (ref. ^30^). The participants provided self-reported information on demographics, lifestyle and disease history via questionnaire and underwent physiological measurements, including providing a blood sample for genetics data. Hospital inpatient data were sourced from the Hospital Episode Statistics for England, Scottish Morbidity Record and Patient Episode Database for Wales, which contain electronic medical records (that is, ICD-10 codes) for all hospital admissions in England, Scotland and Wales, respectively. The hospital inpatient data were available through 31 October 2022 for England, 31 August 2022 for Scotland and 31 May 2022 for Wales. Death register data contained all deaths in the population through 30 November 2022, including primary and contributory causes of death. Participants in both UK Biobank and FinnGen have not received compensation for their participation.

### Assessment of frailty

The HFRS was calculated according to a previously described protocol^4^ based on 109 weighted ICD-10 codes. The codes were selected through a data-driven approach to include codes that were most prevalent in individuals with frailty and high healthcare resource use^4^. Each code was assigned with a weight ranging from 0.1 to 7.1, based on its association with frailty and predictive value for frailty-related outcomes^4^. The weights of all relevant ICD-10 codes present in an individual’s records were then extracted and summed to calculate the HFRS score. The conditions, their respective weights and proportion of individuals with each condition in FinnGen and the UK Biobank are listed in Supplementary Table 18. The HFRS was used as a continuous variable in the GWAS. We also categorized the HFRS into low (<5), intermediate (5–15) and high (>15) risk of frailty as previously described^4^ and used the cutoff points to describe frailty in our study populations. In the main analysis, we included all available ICD-10 codes for each person from age 30 years to the age at the end of follow-up to calculate the HFRS. As dementia diagnoses have the highest weight in the HFRS, we also calculated the HFRS by excluding dementia weights from the formula and performed sensitivity analyses on all analyses using the HFRS without dementia.

### Genotyping and imputation

Genotyping in FinnGen was performed in Illumina and custom AxiomGT1 Affymetrix (Thermo Fisher Scientific) genome-wide arrays and imputed to 16,387,711 (imputation INFO score > 0.6) variants using a population-specific SISu v.3 imputation reference panel as previously described^31^. Individuals with ambiguous sex and non-Finnish ancestry were excluded. UK Biobank samples (v3 genotyping release) were genotyped on custom Affymetrix microarrays and imputed using the 1000 Genomes and the Haplotype Reference Consortium reference panels to ~93 million variants^32^. Participants were excluded if they were flagged as having unusually high heterozygosity or missing genotype calls (<5%). Our analysis was restricted to participants with European descent and white British ancestry (*N* = 407,463). Detailed procedures on genotype calling, quality controls and imputation have been previously described for FinnGen^29^ and the UK Biobank^32^.

### Statistics and reproducibility

No statistical method was used to predetermine sample size, as the UK Biobank and FinnGen cohorts are sufficiently large and can be anticipated to provide adequate statistical power for the planned analyses. We have sought to include all samples after exclusion based only on incomplete data, such as sex, birth year and genotype quality control as called by the respective cohorts. In the case of the UK Biobank, non-European descent and non-white British ancestry participants were excluded to facilitate the comparison to the homogeneous FinnGen Finnish populations. Our study did not involve randomization/allocation into experimental groups, as it was an observational, hypothesis-free GWAS treating the HFRS as a continuous outcome. Therefore, no experimental manipulation or group assignment was performed. In a hypothesis-free GWAS, blinding is not possible/necessary as the analysis is fully automated and applies standardized statistical tests uniformly across the genome. Data distribution was assumed to be normal, but this was not formally tested.

### Discovery GWAS, replication and meta-analysis

The analytical pipeline for GWAS and post-GWAS analyses is presented in Fig. 1. We first performed GWASs of HFRS and HFRS without dementia in FinnGen using the SAIGE^33^ (v.0.35.8.8) software, which uses linear mixed-effects modeling to account for genetic relatedness and confounding by ancestry^34^. We included variants (*N* = 21,294,561) with minor allele frequency > 0.01%, Hardy–Weinberg *P* value > 1 × 10^−9^ and imputation INFO score ≥ 0.9. The models were adjusted for birth year, sex and the first ten PCs. The genome-wide significance level was set to 5 × 10^−8^. The total number of genes to which the variants were mapped was determined by extracting variants with a *P* < 5 × 10^−8^, followed by variant mapping and annotation using the Variant Effect Predictor^35^ in the standard FinnGen GWAS annotation pipeline^29^. Independent lead variants were identified using the R package gwasRtools^36^. We used a distance-based loci definition on the genome-wide significant variants (that is, 500 kb from index variant) to estimate the independent genomic loci. Independent lead variants were identified by linkage disequilibrium clumping and defined as those that were independent from each other at *r*^2^ < 0.01.

To replicate the findings at the variant level, we performed both HFRS GWASs in the UK Biobank. To account for the related samples in the UK Biobank, we applied a mixed linear model-based GWAS analysis (‘fastGWA’)^37^, which is an efficient method to control for relatedness by a sparse genetic relationship matrix, without the need of excluding related individuals. The models were adjusted for birth year, sex, genotyping array and the first ten PCs. Finally, to capture the totality of the evidence, we conducted a meta-analysis on the results from FinnGen and the UK Biobank using METAL^38^. A fixed-effect meta-analysis was performed using the default approach, with *P* value and direction of effect weighted according to sample size, and with adjustment for genomic control (lambda). Using the NHGRI-EBI GWAS Catalog^39^ filtered for *P* < 5 × 10^−8^ and results of previous GWASs into frailty (using the FP^11^ and FI^10^ to measure frailty) and mvAge^13^, a genomic structural equation modeling-derived composite construct of healthspan, parental lifespan, extreme longevity, frailty and epigenetic aging, we assessed the number of novel and previously unreported associations relative to the FinnGen results.

### Genetic correlation and heritability

Using linkage disequilibrium score regression^40^ (v1.0.1) and linkage disequilibrium merged with the HapMap3 reference panel of ~1.1 million variants, we estimated (1) the potential bias from, for example, population stratification and cryptic heritability in the GWAS results, (2) heritability of HFRS and (3) genetic correlations between HFRS and previous GWASs of FI^10^, FP^11^ and mvAge^13^. As the FI GWAS^10^ used an opposite effect allele compared to the standard FinnGen workflow, we inverted the genetic correlation coefficient to prevent an artifactual negative correlation and facilitate interpretation.

### Functional annotation: cell-type and pathway enrichment

To explore tissue and cell-type specificity of the annotated genes underlying HFRS, we applied WebCSEA, a web platform to derive context-specific expression patterns of genes underlying complex traits, encompassing the Human Cell Atlas and single-cell data resources^41,42^. Enrichr pathway analysis^14^ based on KEGG^39^ and Reactome^40^ resources was applied to explore enriched pathways of the identified genes (GWAS *P* < 5 × 10^−8^). To effectively compare the enriched pathways of the HFRS with those of the FI and FP GWASs, we extracted all genome-wide significant variants from these GWASs and performed KEGG and Reactome pathway analyses using the same (default) settings.

### Proteomics integration

To prioritize genes and identify potentially functional and causal variants, we narrowed down the association signals to a smaller number of missense, splice region, loss of function and 5′ and 3′ untranslated region variants (the two last mentioned potentially affecting transcript stability, localization and signal response), identified from the Variant Effect Predictor pipeline^35^, that were associated with the HFRS at a slightly more relaxed threshold (*P* < 5 × 10^−7^). Using the Olink proteomics data, we then examined if the protein levels of the variants (at a gene-level resolution) were associated with HFRS in the UK Biobank. Details of the UK Biobank Olink proteomics assay, quality-control and data processing procedures have been described elsewhere^43^. Briefly, 54,239 UK Biobank participants were selected for the proteomics profiling using EDTA plasma samples collected at the baseline assessment. Of the 54,239 samples, 46,595 were randomly selected, while 6,376 were chosen by UKB-PPP consortium members and 1,268 were from participants in the coronavirus disease 2019 repeat imaging study, resulting in a sample that was predominantly, but not entirely, random. A total of 2,923 proteins were measured across 8 protein panels using the antibody-based Olink Explore 3072 platform. Protein levels were measured in Normalized Protein eXpression values, which represent the relative concentration of proteins on a log_2_ scale. All the protein levels were scaled to mean = 0 and s.d. = 1 before the association testing. Linear regression models were then performed to assess the associations between the proteins that were available in the Olink platform and HFRS, adjusting for (i) birth year, sex and the first ten PCs and (ii) batch, baseline assessment centers, BMI and smoking. We considered an FDR < 0.05 as statistically significant in the proteomics analysis.

### Colocalization analyses

To further prioritize the genes and identify causal variants, we performed a Bayesian-based colocalization analysis with eQTL, sQTL and pQTL, using a flanking window of 1 Mb and default parameters for prior probabilities^12^. The analysis assumes that only one causal variant exists for each trait in a genomic locus and returns PPs indicating the likelihood that the following hypotheses (H) are true: there is no association at the locus with either expression/splicing/protein level or HFRS (H0); there is an association with expression/splicing/protein level but not HFRS (H1); there is no association with expression/splicing/protein level, but there is an association with HFRS (H2); there is an association with both expression/splicing/protein level and HFRS, but with distinct causal variants (H3); there is an association with both expression/splicing/protein level and HFRS with a shared causal variant (H4). We considered the analysis having enough power if the sum PPs had a distinct or shared causal variant exceeded 88%. A colocalized signal was detected if the PP of a shared causal variant (H4) existence was greater than 80%. The GTEx database^44^ (v8) was interrogated for eQTL and sQTL, while the UK Biobank Pharma Proteomics Project^43^ was used for pQTL.

### PRS analyses

Using the GWAS summary statistics from FinnGen, we calculated the PRSs for HFRS in the UK Biobank by applying PRSs with continuous shrinkage^45,46^ and using the European panel from the 1000 Genomes^46^ linkage disequilibrium reference, where ~1.1 million variants were selected. Using linear regression, we fitted a linear model to assess how the HFRS-PRSs associate with the HFRS score. HFRS was considered as a standardized *z*-score in the linear regressions. We also performed logistic regressions to assess the associations of the HFRS-PRSs with early-onset frailty, defined as HFRS > 5 before age 65. Age 65 was chosen as the cutoff as it is commonly used to distinguish ‘young’ from ‘old’ in statistical and policy contexts. Our previous work also identified age 65 as the optimal threshold for distinguishing between early-life and late-life frailty^47^. The PRS was modeled per standard deviation change, and all the models included birth year, sex and the first ten PCs as covariates.

Lastly, as frailty manifests in late life for most individuals, we asked whether the HFRS-PRSs could be used in early risk stratification to identify individuals at risk of adverse outcomes. To this end, Cox models with attained age as the timescale and linear regression models were fitted to assess whether the HFRS-PRSs predict all-cause mortality and number of hospitalizations, respectively. The added value of the HFRS-PRSs beyond age and sex in the prediction was assessed using the *F*-test for linear regressions and likelihood-ratio test for Cox models. The number of hospitalizations was scaled to a mean = 0 and s.d. = 1 before modeling.

### Prediction of mortality using HFRS

Cox models with attained age as the timescale, which inherently adjusts for age, were fitted to assess the association between HFRS, HFRS without dementia and all-cause mortality in FinnGen and the UK Biobank. Two models were fitted for each HFRS definition: one adjusting for sex and birth year, and one without adjustments.

### Ethics statements of FinnGen and UK Biobank

#### FinnGen

Patients and control participants in FinnGen provided informed consent for biobank research, based on the Finnish Biobank Act. Alternatively, separate research cohorts, collected before the Finnish Biobank Act came into effect (in September 2013) and the start of FinnGen (August 2017), were collected based on study-specific consents and later transferred to the Finnish biobanks after approval by the Finnish Medicines Agency, the National Supervisory Authority for Welfare and Health. Recruitment protocols followed the biobank protocols approved by the Finnish Medicines Agency. The Coordinating Ethics Committee of the Hospital District of Helsinki and Uusimaa (HUS) statement number for the FinnGen study is HUS/990/2017. The FinnGen study is approved by Finnish Institute for Health and Welfare (permit nos. THL/2031/6.02.00/2017, THL/1101/5.05.00/2017, THL/341/6.02.00/2018, THL/2222/6.02.00/2018, THL/283/6.02.00/2019, THL/1721/5.05.00/2019 and THL/1524/5.05.00/2020), Digital and population data service agency (permit nos. VRK43431/2017-3, VRK/6909/2018-3 and VRK/4415/2019-3), the Social Insurance Institution (permit nos. KELA 58/522/2017, KELA 131/522/2018, KELA 70/522/2019, KELA 98/522/2019, KELA 134/522/2019, KELA 138/522/2019, KELA 2/522/2020 and KELA 16/522/2020), Findata (permit nos. THL/2364/14.02/2020, THL/4055/14.06.00/2020, THL/3433/14.06.00/2020, THL/4432/14.06/2020, THL/5189/14.06/2020, THL/5894/14.06.00/2020, THL/6619/14.06.00/2020, THL/209/14.06.00/2021, THL/688/14.06.00/2021, THL/1284/14.06.00/2021, THL/1965/14.06.00/2021, THL/5546/14.02.00/2020, THL/2658/14.06.00/2021 and THL/4235/14.06.00/202), Statistics Finland (permit nos. TK-53-1041-17, TK/143/07.03.00/2020 (earlier TK-53-90-20), TK/1735/07.03.00/2021 and TK/3112/07.03.00/2021) and Finnish Registry for Kidney Diseases permission/extract from the meeting minutes on 4 July 2019.

The Biobank Access Decisions for FinnGen samples and data utilized in FinnGen Data Freeze 9 include: THL Biobank BB2017_55, BB2017_111, BB2018_19, BB_2018_34, BB_2018_67, BB2018_71, BB2019_7, BB2019_8, BB2019_26, BB2020_1, Finnish Red Cross Blood Service Biobank 7.12.2017, Helsinki Biobank HUS/359/2017, HUS/248/2020, Auria Biobank AB17-5154 and amendment no. 1 (17 August 2020), AB20-5926 and amendment no. 1 (23 April 2020) and it’s modification (22 September 2021), Biobank Borealis of Northern Finland_2017_1013, Biobank of Eastern Finland 1186/2018 and amendment 22 § /2020, Finnish Clinical Biobank Tampere MH0004 and amendments (21 February 2020 and 06 October 2020), Central Finland Biobank 1-2017, and Terveystalo Biobank STB 2018001 and amendment 25 August 2020.

#### UK Biobank

The UK Biobank study was approved by the North West Multi-centre Research Ethics Committee (approval no. 11/NW/03820). All participants provided written informed consent for data collection, analysis and record linkage. We have also obtained ethical approval for the use of UK Biobank data in Sweden (2016/1888-31/1).

### Reporting summary

Further information on research design is available in the Nature Portfolio Reporting Summary linked to this article.

## Supplementary information

Supplementary Information Supplementary Tables 1–19.

Reporting Summary

## Data Availability

Individual-level data cannot be stored in public repositories or otherwise made publicly available due to ethical and data protection restrictions. However, data are available upon request for researchers who meet the criteria for access to confidential data. Data from the UK Biobank are available to bona fide researchers upon application at https://www.ukbiobank.ac.uk/enable-your-research/. The following UK Biobank-associated data were accessed through, and as part of, our UK Biobank accession: Hospital Episode Statistics for England, Scottish Morbidity Record and Patient Episode Database for Wales. FinnGen results, according to the FinnGen consortium agreement, are subjected to a one-year embargo, and summary statistics are then made available to the scientific community and released two times a year. Information on accessing FinnGen data can be found at https://www.finngen.fi/en/access_results/. The national healthcare, population and cause of death registers were accessed through, and as part of, our FinnGen accession, implemented in the FinnGen pipelines.

## Code availability

All the data processing, visualization and statistical analyses were performed using Python 3.8 (2.7 for LDSC) and R v.4.3.2 (R Foundation for Statistical Computing, Vienna, Austria; https://www.r-project.org/). Meta-analyses were performed using METAL v.2011-03-25. Independent Genomic loci were identified using the R package gwasRtools^36^ (version 0.1.7; https://lcpilling.github.io/gwasRtools/). Venn diagrams were created using the R package ggvenn (version 0.1.10; https://cran.r-project.org/web/packages/ggvenn/index.html). Correlation plots were created using the R package corrplot (v.0.92; https://cran.r-project.org/web/packages/corrplot/index.html). Forest plots were created using the R package ggforestplot (v.0.1.0; https://nightingalehealth.github.io/ggforestplot/). The analysis codes are available as follows: FinnGen GWAS via https://github.com/FINNGEN/saige-pipelines/; UK Biobank GWAS via http://cnsgenomics.com/software/gcta/#fastGWA and QTL colocalization analysis via https://github.com/Moritz-JD-Krueger/Colocalization-Analysis/.
